# Early Auditory Experience Modifies Neuronal Firing Properties in the Zebra Finch Auditory Cortex

**DOI:** 10.3389/fncir.2020.570174

**Published:** 2020-10-08

**Authors:** Takashi Kudo, Yuichi Morohashi, Yoko Yazaki-Sugiyama

**Affiliations:** ^1^Neuronal Mechanism of the Critical Period Unit, Okinawa Institute of Science and Technology (OIST) Graduate University, Okinawa, Japan; ^2^International Research Center for Neurointelligence (IRCN), Institutes of Advanced Studies, The University of Tokyo, Tokyo, Japan

**Keywords:** auditory experience, song learning, songbird, auditory cortex, critical period, zebra finch, firing properties

## Abstract

Songbirds learn to sing much as humans learn to speak. In zebra finches, one of the premier songbird models, males learn to sing for later courtship through a multistep learning process during the developmental period. They first listen to and memorize the song of a tutor (normally their father) during the sensory learning period. Then, in the subsequent sensory-motor learning phase (with large overlap), they match their vocalizations to the memorized tutor song *via* auditory feedback and develop their own unique songs, which they maintain throughout their lives. Previous studies have suggested that memories of tutor songs are shaped in the caudomedial nidopallium (NCM) of the brain, which is analogous to the mammalian higher auditory cortex. Isolation during development, which extends the sensory learning period in males, alters song preference in adult females, and NCM inactivation decreases song preference. However, the development of neurophysiological properties of neurons in this area and the effect of isolation on these neurons have not yet been explained. Here, we performed whole-cell patch-clamp recording on NCM neurons from juvenile zebra finches during the sensory learning period, 20, 40, or 60 days post-hatching (DPH) and examined their neurophysiological properties. In contrast to previous reports in adult NCM neurons, the majority of NCM neurons of juvenile zebra finches showed spontaneous firing with or without burst firing patterns, and the percentage of neurons that fired increased in the middle of the sensory learning period (40 DPH) and then decreased at the end (60 DPH) in both males and females. We further found that auditory isolation from tutor songs alters developmental changes in the proportions of firing neurons both in males and females, and also changes those of burst neurons differently between males that sing and females that do not. Taken together, these findings suggest that NCM neurons develop their neurophysiological properties depending on auditory experiences during the sensory song learning period, which underlies memory formation for song learning in males and song discrimination in females.

## Introduction

Songbirds learn to sing by imitating adult conspecific vocalizations during development in a way that resembles how humans learn to speak. In zebra finches, in which only male birds sing for courtship, a bird’s song is sculpted during the developmental critical period and is then retained throughout its entire life. Male juvenile zebra finches first listen to and memorize their tutor’s (normally their father’s) songs during the sensory learning period and then match their vocalizations to the memorized tutor songs using auditory feedback in the subsequent (with a large overlap) sensory-motor learning period. Tutor song experiences sculpt neuronal circuits of juvenile songbirds by forming memories of tutor songs. Formed memories of tutor songs are thought to guide later motor song learning in males (Brainard and Doupe, 2002; Bolhuis and Gahr, 2006; Theunissen and Shaevitz, 2006) and also to affect auditory cognitive functions of females in later auditory learning (Chen et al., 2017; Diez et al., 2019). Accumulating evidence suggests that memories of tutor songs for male juvenile song learning are formed in the caudomedial nidopallium (NCM), which is analogous to the mammalian higher auditory cortex, while other studies have suggested the presence of tutor song memories in the song motor area (Mackevicius and Fee, 2018; Zhao et al., 2019). A greater number of NCM neurons express the immediate early gene, ZENK, in zebra finches that are exposed to experienced tutor songs than in those that are exposed to novel conspecific songs (Terpstra et al., 2004). Moreover, blockade of extracellular signal-regulated kinase activity in the NCM during tutor song exposure resulted in less learning from tutor songs (London and Clayton, 2008). Electrophysiological evidence has further shown that NCM neuronal auditory responses habituated more rapidly when adult head-restrained birds listened to novel songs than when they repeatedly listened to tutor songs (Phan et al., 2006). Recently, we showed that a specific fraction of NCM neurons in juveniles responded exclusively to tutor songs after learning from tutors in freely behaving conditions, and this selectivity was reduced by blocking local GABA inhibitory function, suggesting that early auditory experiences shape NCM neuronal circuits to form tutor song memories by recruiting GABA inhibitory circuits (Yanagihara and Yazaki-Sugiyama, 2016). While fewer studies of NCM function in female auditory behavior have been reported, recent studies have shown that transient inactivation of the NCM caused decreased specificity in female song preferences (Tomaszycki and Blaine, 2014) and that the NCM neuronal response to song stimulation can also be modulated with song experiences in early life (Diez et al., 2019).

The NCM receives auditory inputs from the primary auditory cortex, L2a (Vates et al., 1996; Mello et al., 1998), and the local inhibitory network shapes auditory responsiveness (Pinaud et al., 2008). In the mammalian auditory cortex, mIPSCs develop depending upon auditory experiences during development. Hearing loss at an early stage, but not later, delays the sharpening of the decay time of mIPSCs (Takesian et al., 2013). In zebra finches, adult NCM neurons in both sexes were mostly neurophysiologically silent (Dagostin et al., 2015), and adult auditory experiences modified the probabilities of burst glutamatergic currents (Dagostin et al., 2012). However, intrinsic properties, including spontaneous activity of NCM neurons and other changes during the sensory learning period resulting from auditory experiences, have not yet been examined, especially in females.

To examine neuronal activity in the NCM during the sensory learning period, we performed whole-cell patch-clamp recordings from brain slices from male and female juvenile zebra finches. Here, we first reported that most NCM neurons from juvenile zebra finches of both sexes show spontaneous firing, in contrast to previously reported findings from adult NCM neurons (Dagostin et al., 2015), which were subsequently separated into burst- or non-burst-type neurons. We further found that the proportions of firing neurons changed depending on auditory experiences during sensory development. Interestingly, the developmental trajectory of neuronal type proportions and effects of auditory experiences on those trajectories were found to differ between juvenile males and females.

## Materials and Methods

### Animals

Experiments were performed following experimental protocols approved by the animal care committee at Okinawa Institute of Science and Technology (OIST) Graduate University. Sixty-seven male and 69 female zebra finches, hatched and reared in our colony (88 normally reared and 48 isolated), were used. The normally reared birds were raised in cages with their parents and siblings until they were sacrificed for experiments at 20, 40, or 60 days post-hatching (DPH). Juveniles to be isolated were raised by their parents until 10–12 DPH when their fathers were removed, and the juveniles were subsequently raised with their mothers and siblings in a cage in a sound-attenuating chamber until they were sacrificed for experiments at 20, 40, 60, or 80 DPH. The sex of juvenile birds was checked by PCR around 20 DPH. We did not monitor the singing behavior of either normally reared or isolated juveniles.

### Brain Slice Preparations

Zebra finches were deeply anesthetized with isoflurane (Wako, Osaka, Japan) and then rapidly decapitated. Brains were extracted from the skulls and placed in chilled low-Ca^2+^ cutting solution [in mM: 200 sucrose, 2.5 KCl, 10 glucose, 1.25 NaH_2_PO_4_, 2 Na pyruvate, 3 myoinositol, 0.5 ascorbic acid, 26 NaHCO_3_, 1 trehalose, 6 MgCl_2_, 0.1 CaCl_2_; pH 7.2–7.4 (290–310 mOsm)], then trimmed into a brain block containing the NCM. Brain blocks were sliced in the sagittal plane with a vibratome (PRO 7; Dosaka EM, Kyoto, Japan) at 400-μm thickness. Slices were kept in aerated (95% O_2_/5% CO_2_) artificial cerebrospinal fluid [ACSF, in mM: 125 NaCl, 2.5 KCl, 10 glucose, 1.25 NaH_2_PO_4_, 2 Na pyruvate, 3 myoinositol, 0.5 ascorbic acid, 26 NaHCO_3_; pH 7.2–7.4 (290–310 mOsm)] at 30°C until incubation at room temperature for at least 0.5 h before being transferred to an electrophysiological recording chamber, as previously described (Yazaki-Sugiyama et al., 2015).

### Whole-Cell Patch-Clamp Recordings

Brain slices were placed in a recording chamber (Scientifica, Uckfield, UK) attached to an upright DIC microscope stage (Scientifica), which allowed visual guidance for whole-cell patch-clamp recording by using IR illumination. We recorded neurons in the NCM by visual inspection, and their locations in the NCM were confirmed later by histological identification. Throughout the experiments, slices were continuously perfused (2 ml/min) with ACSF aerated with 95% CO_2_/5% O_2_. Electrodes (5–8 MΩ) were pulled from glass capillaries (Harvard Apparatus, Holliston, MA, USA) with a multistage puller (Zeitz Instruments, Bayern, Germany) and filled with an internal K-gluconate solution (in mM: 135 K-gluconate, 0.5 CaCl_2_, 2 MgCl_2_, 2 MgATP, 5 EGTA, 10 HEPES, 0.1 GTP). pH and osmolality were adjusted to 7.3 and 300 mOsm, respectively. Previous research used higher osmolarity ACSF with 10 mM NaCl (Bottjer, 2005; Dagostin et al., 2015), but our preliminary experiments did not show different results. Electrodes were lowered to the NCM with visual guidance under a microscope and attached to the vicinity of the membrane while maintaining positive pressure. After forming a high-resistance seal (approximately 2–10 GΩ) by applying negative pressure, the second pulse of negative pressure was used to break the membrane to achieve whole-cell recordings. Junction potentials between the pipette and the extracellular solution were canceled by the voltage offset of the amplifier before establishing a seal and were not further corrected. The liquid junction potential, which was theoretically calculated at 25°C, was 16.0 mV. Recordings were obtained using a MultiClamp 700B amplifier (Molecular Devices, San Jose, CA, USA), digitized at 10 kHz, low-pass filtered at 4 kHz, and monitored online with pClamp (version 10; Molecular Devices), and data were stored on a PC. Spontaneous firing rate (SFR) and resting membrane potentials (RMPs) were recorded five times under no current injections, each of which was a 30-s recording under the current-clamp mode, followed by injection of a 500-ms square step hyperpolarizing pulse current three times (slope of the voltage responses from −10 to −50 pA of the current injection) to calculate the input resistance (Rin). All recordings were performed at room temperature, as NCM neurons do not survive at higher temperatures (>30°C; Dagostin et al., 2015). Series resistance was constantly monitored, and neurons with resistances greater than 30 MΩ or smaller than 5 MΩ were excluded. Cells with holding currents greater than 0 or less than −20 pA, with capacitance greater than 100 pF were also excluded. Active neurons, which showed spontaneous firing, were further separated into burst-type or non-burst-type, based on their interspike intervals (ISIs). Burst-type neurons were defined as showing more than two spikes with less than 30-ms ISI.

### Cell Identification

Recorded neurons were histologically identified *post hoc*. Neurobiotin-Plus (final concentration 0.25%; Vector Labs, Burlingame, CA, USA) was electrophoretically injected through micropipettes into recorded neurons after an electrophysiological examination. Within 10 h after electrophysiological recordings, slices containing Neurobiotin-Plus-filled neurons were fixed by overnight immersion in 4% paraformaldehyde (PFA) in phosphate-buffered saline (PBS) at 4°C. Then, fixed slice tissues were processed using a tissue-clearing method, the CLARITY technique, and Neurobiotin-Plus-filled neurons were immunohistochemically visualized. Slices were incubated in A4P0 hydrogel solution (4% acrylamide, 0.25% VA-044/PBS) at 4°C overnight. Then, the hydrogel A4P0 solution was bubbled with N_2_ gas for 5 min and incubated at 37°C for 3 h to form a hydrogel. After washing with PBS, slices were then incubated with 8% SDS/1 M boric acid buffer with gentle shaking at 37°C for 2 days. After washing with 0.2 M boric acid buffer/0.2% Triton X-100 solution for 1 day, slices were incubated with rabbit polyclonal anti-biotin antibody (Bethyl, Montgomery TX, 1:800 in PBS/0.3% Triton X-100/0.01% NaN_3_) for 1 day at 37°C and then with an Alexa-546-conjugated secondary antibody (ThermoFisher Scientific, Waltham, MA, USA 1:800 in PBS/0.3% Triton X-100/0.01% NaN_3_) for 2 days at 37°C. After washing with PBS, slices were post-fixed in 4% PFA in PBS for 1 h and washed with PBS. Then, slices were incubated with PROTOS to match the refractive index overnight (Murray et al., 2015). Slices were mounted on glass-bottomed dishes and imaged with a confocal microscope with a 20× lens (LSM 780; Carl Zeiss, Oberkochen, Germany).

### Adeno-Associated Virus (AAV) Production and Injection

The AAV of serotype 9 that expressed mRFP1 specifically in inhibitory neurons using the mDlx promoter (Dimidschstein et al., 2016; AAV9-mDlxTetOff-mRFP1) was generated by tripartite transfection (AAV-rep2/cap9 expression plasmid, adenovirus helper plasmid, and AAV-vector plasmid) into AAV-293 cells (Agilent, Santa Clara, CA, USA). The pAAV2-mDlx-TetOff-mRFP1 plasmid was constructed from pAAV2-SynTetOff-GFP (Sohn et al., 2017) by replacing the promoter and GFP coding sequences with a GABAergic neuron-specific mDlx promoter (Dimidschstein et al., 2016) and mRFP1 coding sequences, respectively. AAV9-mDlxTetOff-mRFP1 was bilaterally injected into the NCM (184–276 nL) of normally raised juvenile zebra finch brains (44–47 DPH) through a pipette connected to a pressure injector (Nanoject II; Drummond Scientific Company, Broomall, PA, USA) with stereotaxic coordination under isoflurane anesthesia (2.0–3.0%). After approximately 2 weeks of survival (59–60 DPH), AAV-injected juveniles were sacrificed for whole-cell patch-clamp recordings using methods described above.

### Experimental Design and Statistical Analysis

Data were collected from five male and 11 female juveniles at 20 DPH (18–20 DPH, 22 and 26 neurons, respectively), 18 male and 14 female juveniles at 40 DPH (39–41 DPH, 26 and 24 neurons, respectively), and 16 male and 14 female juveniles at 60 DPH (59–61 DPH, 24 and 23 neurons, respectively). All were normally reared birds. Neuronal activity was also recorded from brain slices of two male and three female juveniles isolated at 20 DPH (22–25 DPH, 10 and 15 neurons, respectively), seven males and nine females at 40 DPH (39–41 DPH, 14 and 13 neurons, respectively), five males and seven females at 60 DPH (60–61 DPH, 33 and 22 neurons, respectively), and 14 males and 6 females at 80 DPH (79–82 DPH, 16 and nine neurons, respectively). The activity of 1–11 neurons from each juvenile bird was recorded. The activity of neurons labeled with mRFP, from two males and eight females, was also recorded at 60 DPH. These birds were injected with AAV9-mDlxTetOff-mRFP1 at ~45 DPH (5 and 14 neurons, respectively). The activity of 1–3 neurons from each juvenile bird that was injected with AAV9-mDlxTetOff-mRFP1 was recorded.

To compare proportions of distinct neural types in the SFR (lower firing, higher firing, and silent) or proportions of distinct burst or non-burst neuronal types at each age, Fisher’s exact test was applied using R (version 3.3.3). We also compare the average SFR of all recorded neurons at each age/sex/rearing condition which was estimated by using a generalized linear model with a Poisson distribution (MatLab, MathWorks, MA, USA). For comparisons of RMPs or Rin between males and females, between ages (20, 40 or 60 DPH) or neuronal types with the SFR (higher firing, lower firing, or silent), we applied three-way ANOVA with age, sex, and neuronal type as factors to perform unbiased analyses of significant interactions and main effects with Tukey *post hoc* tests, using Origin Pro (OriginLab; Northampton, MA, USA). To see histological distribution differences in each type of neuron, we compared the proportions of each neuronal type with SFR or burst type between the ventral or dorsal part of the NCM to that in the total number of neurons using an *X*^2^ test. In isolated birds, the same statistical analyses were performed. All comparisons were considered significantly different if *p* < 0.05. Data are shown as means ± standard errors.

## Results

### Developmental Changes in the Proportion of Neuronal Types in the NCM

**Figure 1:**
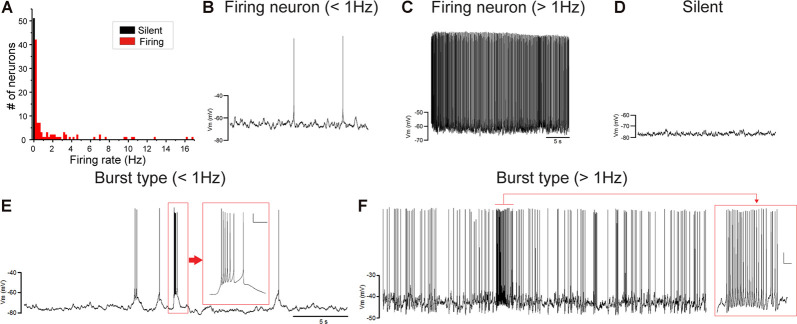
Caudomedial nidopallium (NCM) neurons with or without spontaneous firing. **(A)** A histogram of the number of NCM neurons plotted against their spontaneous firing rates (SFRs) (73 cells from 39 males and 74 cells from 39 females). Fifty-three neurons showed no spontaneous firing. Representative traces of neuronal activities from firing neurons with lower-SFR **(B)** and higher-SFR **(C)** and a silent neuron **(D)**. Firing neurons were further divided into burst-type or non-burst-type neurons, based on interspike intervals (ISIs). Representative traces of burst-type firing in a with lower SFR **(E)** and higher-SFR **(F)** neuron. Inserts: expanded traces from the red square showing burst firing with firing at less than 30 ms ISI (horizontal scale bar, 200 ms; vertical scale bar, 10 mV).

**Figure 2:**
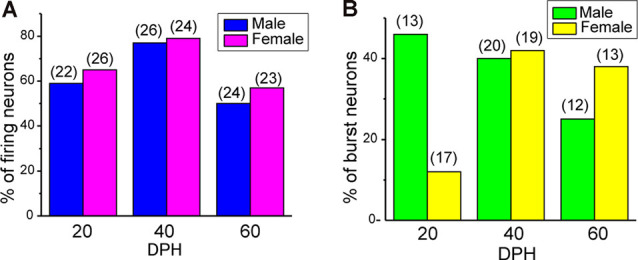
The proportion of neuronal types changes during development. **(A)** Proportions of firing neurons recorded from normally raised male and female juveniles at 20, 40, and 60 days post-hatching (DPH). The number of neurons recorded is shown in parentheses (*N* = 5, 18, and 16 for males at 20, 40, and 60 DPH; 11, 14, and 14 for females at 20, 40, and 60 DPH birds, respectively). **(B)** Proportions of burst-type neurons recorded from normally raised male and female juveniles at 20, 40, or 60 DPH (*N* = 4, 17 and 12 for males at 20, 40, and 60 DPH; 10, 12, and 10 for females at 20, 40 and 60 DPH birds, respectively).

**Figure 3:**
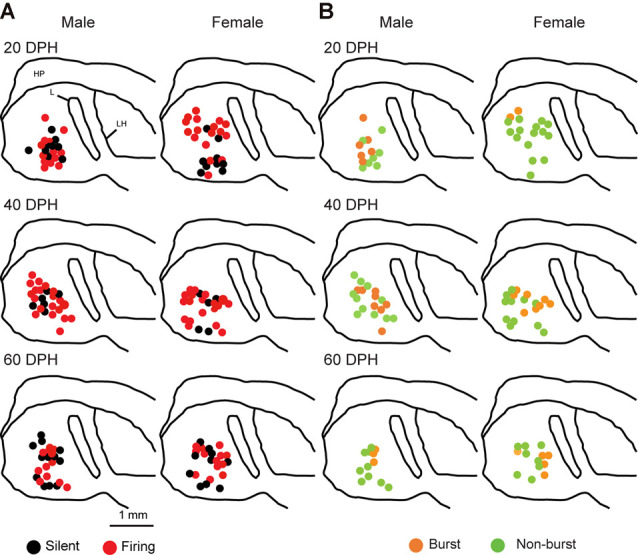
Types of neurons with an SFR and burst-type were not correlated with a location within the NCM (*X*^2^ test). Anatomical locations of recorded neurons are color-coded with the neuronal type with SFR **(A)** or burst-type **(B)** from the male (left) or female (right) juveniles at 20 (top), 40 (middle), or 60 (bottom) DPH in a schematic including the NCM, 400–800 mm from the midline. HP, hippocampus; L, field L; LH, lamina hyperstriatica. B: The anatomical location of burst and non-burst-type NCM neurons.

Juvenile male zebra finches listen to and memorize their tutor songs in the early sensory learning period (20–50 days) for later sensory-motor learning. Memories of tutor songs are thought to be stored in the NCM. In females, early auditory experiences modulate song preferences in adulthood (Chen et al., 2017) and inactivation of the NCM alters song preference (Tomaszycki and Blaine, 2014). ZENK expression in the NCM upon song stimulation can also be modulated by song experiences in early life (Diez et al., 2019). These findings suggested that NCM neuronal activities are shaped during the sensory learning period in both males and females. To understand the basic neurophysiological properties of NCM neurons during the sensory learning period, we performed whole-cell patch-clamp recordings from brain slices of a juvenile male and female zebra finches at 20 (18–20 DPH, 22 and 26 neurons from five males and 11 females, respectively), 40 (39–41 DPH, 26 and 24 neurons from 18 males and 14 females, respectively), and at 60 DPH (59–61 DPH, 24 and 23 neurons from 16 males and 14 females, respectively). We measured the SFR of each NCM neuron and found that the majority of recorded neurons (64%, 94/145 neurons) exhibited spontaneous firing (Figure 1A, data pooled from all age and sex), which contrasted with previous reports in adults, where the majority of NCM neurons showed no spontaneous firing activity (Dagostin et al., 2015). Most of the firing neurons showed a lower firing rate (<1 Hz; Figure 1B), while some others showed a higher (>1 Hz) firing rate (Figure 1C). Proportions of firing neurons in the NCM was 62% (30/48) at 20 DPH and transiently increased to approximately 80% at 40 DPH, the middle of the sensory learning period. Then, by 60 DPH, they decreased to a level comparable to that at 20 DPH in both males and females (Figure 2A). We found that distributions of silent and firing neurons differed significantly between ages (20, 40, 60 DPH, Fisher’s exact test, *p* = 0.0007), while SFR of firing neurons did not differ with age, sex, or those interactions (*p* > 0.05, two-way ANOVA; Supplementary Figure 1A). We also estimated the mean SFR of all the neurons recorded in each sex and age by using a generalized linear model with a Poisson distribution (Supplementary Table 1). The model fitted showed the dependence of the SFR distribution on age and sex (*p* < 0.01). The average RMP were not different with age, sex, or those interactions (Male: 20DPH: −68.56 ± 2.34, 40DPH: −62.43 ± 1.70, 60DPH: −62.15 ± 1.78; Female: 20DPH: −58.15 ± 1.37, 40DPH: −60.23 ± 1.93, 60DPH: −61.35 ± 1.64 (mV), mean ± SEM, >0.05, two-way ANOVA). Taken together those suggest the changes in SFR were not depending on the changes in RMP. We recorded both silent and firing neurons from slices of the same juvenile birds (Supplementary Figure 1C) and found no correlation between the RMP and SFR of recorded neurons (*DF* = 146, *r*^2^ = 0.066; Supplementary Figure 1B). We further noticed that some firing NCM neurons exhibited spontaneous firing bursts (burst-type neurons; Figures 1E,F). We also examined developmental changes in the proportions of burst-type neurons in the NCM during the sensory learning period. Interestingly, the proportion of burst-type neurons increased at 40 DPH only in females (male: 40%, female: 42%) compared to 20 DPH (46% and 12%, respectively) and decreased only in males at 60 DPH (25% and 38%, respectively; Figure 2B). We also investigate the correlation between SFR and burst rates, and that found burst type neurons might be further divided into three groups with their SFR and burst rate combinations (Supplementary Figure 2C). With *post hoc* histological identification of recording sites, we found no distributional differences between the dorsal and ventral parts of the NCM in either type of neuron, silent or firing (Figure 3A) or the burst type (Figure 3B) at any age or sex (*p* > 0.05, *X*^2^ test).

### NCM Neuronal Type Characterization

Most of the firing neurons in male and female juvenile zebra finches (60/94, recorded 20–60 DPH) showed a low firing rate (<1 Hz; Figures 1A,B), while some neurons showed a higher firing rate (>1 Hz; Figure 1C); however, we found no clear distinction within firing neurons with their SFR. SFRs of firing neurons did not differ with age, sex, or their interaction (two-way ANOVA, *p* > 0.05). We also investigated spike half widths of firing neurons and found no correlation with firing rate (*DF* = 92, *r*^2^ = 0.021; Supplementary Figure 2A). We developed neuronal images of recorded neurons (13 silent from seven males and five females and 33 firing neurons from 11 males and 16 females) and found that all neurons that showed higher firing rates (>1 Hz) had aspiny dendrites (two and eight neurons from males and females, respectively), suggesting that higher-firing neurons were inhibitory neurons. However, not all the neurons that had aspiny dendrites showed higher firing rates (>1 Hz). On the other hand, we found eight out of 33 morphologically identified firing neurons were spiny neurons and all of these eight neurons had a lower firing rate (<1 Hz; Supplementary Figure 2B).

**Figure 4:**
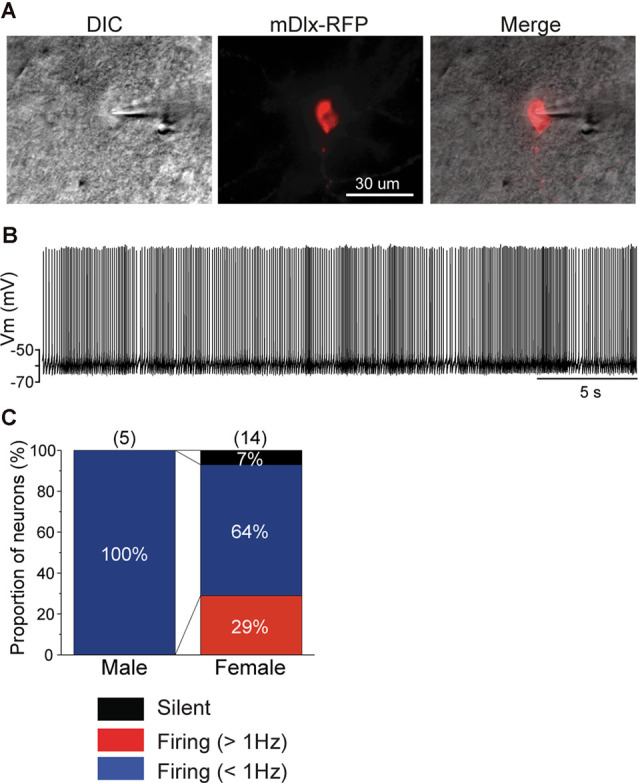
mDlx-RFP-expressing neurons are higher firing neurons in males, but comprise a variety of types in females. **(A)** A DIC image of a representative slice of a patch-clamp recording from an mDlx-RFP-injected juvenile (left), the mDlx-RFP (middle), and merged (right). **(B)** Representative waveforms recorded from an mDlx-RFP-positive neuron. **(C)** Proportions of the three types of GABAergic neurons, separated based on SFR in male and female juveniles at 60 DPH.

Recently, an inhibitory neuron-specific promotor, mDlx, was identified, and it appears to apply to zebra finches (Dimidschstein et al., 2016). To identify neuronal cell types in the NCM, we developed an AAV vector in which we employed the Tet-off system (Sohn et al., 2017) under the mDlx promotor to ensure increased expression of a fluorescent protein (mRFP) specifically in inhibitory neurons. We then performed targeted patch-clamp recording from neurons expressing mRFP in normally raised juveniles at 60 DPH (59–60 DPH) that were injected with AAV-mDlx-mRFP at 46 DPH (44–47 DPH). All recorded neurons that expressed mRFP in males showed more than 1 Hz SFRs (Figures 4A–C), despite a small population of this neuronal type, indicating that GABAergic neurons were higher-firing neurons in males. In contrast, mRFP-expressing neurons recorded from female juveniles showed a variety of firing properties (silent: 7%, <1 Hz SFR: 64%, >1 Hz SFR: 29%; Figure 4C), indicating that not all inhibitory neurons showed higher firing rates in females. mRFP-expressing neurons both males and females had shorter spike half widths (<2.5 ms), especially in males (<1.5 ms; Supplementary Figure 2B).

The RMPs of firing neurons were higher than those of previously reported adult NCM neurons, which showed no spontaneous firing, while those of silent neurons recorded here were comparable. Neurons with higher firing rates were suggested to be inhibitory neurons. With data above, we separated neurons into three groups, silent, lower-, and higher-firing (>1 Hz) neurons with SFRs (Figures 1B,D), and further compared RMPs and Rins, which were measured using stepwise current injections (Supplementary Figure 3), across age, sex, and neuronal type with SFR (Table 1). We found that RMPs were significantly different between ages and neuronal types (three-way ANOVA; age: *F* = 3.40, *DF* = 2, *P* = 0.037; neuronal type: *F* = 17.42, *DF* = 2, *p* = 2.18 × 10^−7^). *Post hoc* analysis with the Tukey test further indicated significant differences in RMPs in each cell type comparison (silent vs. lower, lower vs. higher, and silent vs. higher) as well as the interactions of male silent and higher-firing neurons and female silent and higher-firing neurons (*p* < 0.05), suggesting different neuron types. In comparisons of Rin, we found significant differences within sex and age combinations (*F* = 7.48, *DF* = 2, *p* = 8.71 × 10^−4^, a three-way ANOVA), but not in other factors and their combinations. The *post hoc* Tukey test indicated no differences between any combinations within types of neurons with SFR (*p* > 0.05), suggesting that firing neurons included at least two different cell types, excitatory and inhibitory neurons and that neurons showing higher firing, were inhibitory neurons.

**Table 1:** Resting membrane potentials (RMPs) and input resistances of each neuronal type at each age and sex in normally raised juveniles.

	Resting membrabe potential (RMP)					
	20 DPH		40 DPH		60 DPH	
	M	F	M	F	M	F
Silent	−62.5 ± 4.68 (9)	−64.0 ± 1.62 (9)	−71.2 ± 2.73 (10)	−66.4 ± 5.56 (5)	−63.9 ± 3.26 (12)	−66.6 ± 3.29 (10)
Lower firing	−57.7 ± 2.01 (10)	−57.1 ± 3.29 (7)	−62.2 ± 1.81 (14)	−59.4 ± 2.61 (13)	−61.8 ± 2.86 (7)	−59.5 ± 1.39 (9)
Higher firing	−49.7 ± 5.79 (3)	−53.7 ± 0.80 (10)	−55.4 ± 2.39 (7)	−56.9 ± 1.81 (6)	−58.5 ± 2.20 (5)	−52.0 ± 2.88 (4)
	**Input resistance (Rin)**					
Silent	195 ± 53 (9)	236 ± 65 (9)	416 ± 119 (10)	227 ± 45 (5)	229 ± 33 (11)	441 ± 13 (10)
Lower firing	209 ± 63 (10)	379 ± 12 (7)	495 ± 76 (14)	333 ± 43 (13)	256 ± 48 (7)	346 ± 108 (8)
Higher firing	156 ± 23 (3)	601 ± 84 (10)	560 ± 17 (7)	309 ± 63 (5)	403 ± 30 (3)	387 ± 90 (3)

*RMPs between silent vs. lower, lower vs. higher, and silent vs. higher neurons are significantly different (three-way ANOVA, *post hoc* Tuckey test, *p* < 0.05)*.

### Auditory Isolation Modulates the Development of NCM Neuronal Activity

**Figure 5:**
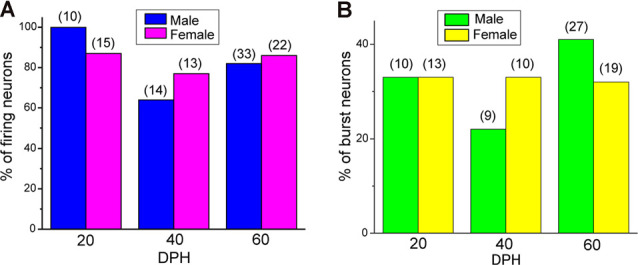
Isolation alters developmental change in the proportions of the type of neurons. Proportions of firing neurons recorded from male and female isolated juveniles at 20, 40, and 60 DPH **(A)**, and proportions of burst- and non-burst-type neurons **(B)** in isolated male and female juveniles at 20, 40, and 60 DPH.

**Figure 6:**
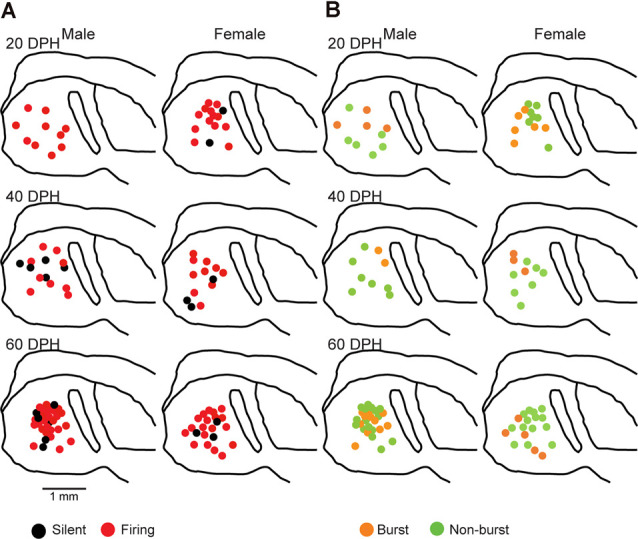
Types of neurons with an SFR and burst type were not correlated with a location in the NCM in isolated juveniles (*X*^2^ test). Anatomical locations of recorded neurons are color-coded with neuronal type with SFR **(A)** or burst type **(B)** from the male (left) or female (right) juveniles at 20 (top), 40 (middle), or 80 (bottom) DPH in a schematic including the NCM, 400–800 mm from the midline.

Proportions of NCM firing and burst type neurons changed over the sensory learning period. The NCM has been suggested to be involved in song learning in males, especially as one of the sites for storing tutor song memories (Bolhuis and Gahr, 2006; Hahnloser and Kotowicz, 2010; Bolhuis and Moorman, 2015), while other brain areas in the song system have also been suggested as tutor song memory loci (Mackevicius and Fee, 2018; Zhao et al., 2019). On the other hand, in females, the NCM is thought to be involved in song discrimination (Tomaszycki and Blaine, 2014). Auditory isolation from adult songs extends the sensory learning period in males (Livingston et al., 2000; Yazaki-Sugiyama et al., 2015) while altering song preferences (Chen et al., 2017) and NCM song responses (Diez et al., 2019) in females. If neurophysiological developments in the NCM were related to auditory song learning or song response behavior, the development of NCM neurons would depend on auditory experiences with tutor songs. Next, we examined whether developmental changes in proportions of NCM neuronal subtypes regarding their firing activities were dependent on auditory song experiences, by performing patch-clamp recording from brain slices of isolated juveniles. These juveniles were isolated from their fathers and reared with their mothers and siblings starting at 10–12 DPH, which prevented them from learning their fathers’ songs (Eales, 1985), while they can hear their own, sibling and female vocalizations, including immature songs. In isolated male juveniles, there were no silent neurons at 20 DPH and the proportion of firing neurons decreased to 64% at 40 DPH, which was comparable to the level in normally reared juveniles at 20 DPH (57%). Then it increased at 60 DPH (82%), similar to the level at 40 DPH in normally reared juveniles (77%; Figure 5A). As there was a delay in the decrease of proportions of firing neurons in isolated male birds, compared to normally raised male birds, we recorded from isolated juveniles at 80 DPH to compare with normally raised birds at 60 DPH. In isolated male birds, the proportion of firing neurons remained high until 80 DPH (75%, Supplementary Figure 4A), unlike normally raised male birds at 60 DPH (47%). A similar trend, a decrease and then an increase in the proportion of firing neurons from 20 to 40 to 60 DPH, remaining high at 80 DPH, was observed in isolated female birds (40 DPH: 77%, 60 DPH: 86% 80 DPH: 89%, Supplementary Figure 4A). SFRs of firing neurons in isolated birds did not differ with age, sex, or their interactions (two-way ANOVA, *p* > 0.05; Supplementary Figure 4C). We found no effect of isolation on SFRs of firing neurons as well as age and sex (three-way ANOVA, *p* > 0.05). We found both silent and firing neurons in brain slices of the same juvenile birds (Supplementary Figure 4E). We also estimated the mean SFR of all the neurons recorded in each sex and age by using a generalized linear model with a Poisson distribution (Supplementary Table 1). The model fitted showed the dependence of the SFR distribution only on age in the isolated birds. We compared proportions of neuronal types, silent and firing neurons between normally reared and isolated birds of the same age (only between 20–60 DPH) and sex and found a significant difference in males at 20 and 60 DPH (*p* = 0.000039 and *p* = 0.048, respectively, Fisher’s exact test). We developed neuronal images of recorded neurons (five silent from three and two from isolated male and females, and nine firing neurons from two and seven isolated males and females) and found that one higher firing neurons (>1 Hz) had spiny dendrites in contrast to normally raised juveniles (Supplementary Figure 4D). We also analyzed firing neurons from isolated juveniles for their burst firing properties. We found that in males, the proportion of burst-type neurons decreased from 20 DPH to 40 DPH, as seen in normally raised birds, but that it increased from 40 to 60 DPH, unlike normally raised birds. On the other hand in females, the proportion of burst-type neurons did not show much change during development, unlike normally raised birds (Figure 5B). There were no distributional differences between the dorsal and ventral parts of the NCM in either type of neuron, based on firing (Figure 6A) or burst-type properties (Figure 6B) at any age or in either sex in isolated juvenile birds (*p* > 0.05, *X*^2^ test).

As in brain slices from normally reared birds, we found that most firing neurons showed less than 1-Hz firing rates, while others had higher firing rates with no clear distinction (Supplementary Figure 4E). RMPs between neuronal types, silent, lower and higher firing rate, were significantly different (three-way ANOVA, *F* = 10.84, *DF* = 2, *p* = 4.76 × 10^−5^), and the *post hoc* Tuckey test indicated a significant difference between male silent and higher firing neurons, as well as between silent and lower, and silent and higher firing neurons in females. On the other hand, we found no differences in Rin between ages, sexes, neuronal types, or any other pairs of metrics (*p* > 0.05, three-way ANOVA; Table 2).

**Table 2:** Resting membrane potentials and input resistances of each neuronal type at each age and sex in isolated juveniles.

	Resting membrabe potential (RMP)					
	20 DPH		40 DPH		60 DPH	
	M	F	M	F	M	F
Silent		−67.8 ± 1.37 (2)	−68.2 ± 5.13 (5)	−60.9 ± 5.45 (3)	−62.5 ± 2.83 (6)	−65.0 ± 5.99 (3)
Lower firing	−54.0 ± 4.50 (2)	−55.4 ± 1.65 (10)	−59.5 ± 2.57 (9)	−52.3 ± 2.62 (4)	−57.8 ± 2.08 (18)	−53.6 ± 1.98 (14)
Higher firing	−53.0 ± 2.03 (8)	−50.3 ± 5.17 (3)		−54.1 ± 1.97 (6)	−56.4 ± 0.94 (9)	−53.2 ± 4.13 (5)
	**Input resistance (Rin)**					
Silent		533 ± 46 (2)	368 ± 41 (5)	691 ± 89 (3)	385 ± 62 (6)	387 ± 44 (3)
Lower firing	404 ± 14 (2)	336 ± 53 (10)	403 ± 55 (7)	438 ± 81 (3)	463 ± 71 (17)	426 ± 85 (14)
Higher firing	397 ± 47 (8)	492 ± 92 (3)		892 ± 23 (6)	284 ± 21 (9)	416 ± 62 (5)

## Discussion

In this study, we examined the spontaneous firing activity of NCM neurons in zebra finch juveniles during the sensory learning period, using whole-cell patch-clamp recordings from slice preparations. Surprisingly, we found much higher proportions of firing neurons in the developing zebra finch NCM in both sexes, compared to previously reported proportions from the adult brain (Dagostin et al., 2015). We cannot completely preclude the possibility of differences in recording conditions, such as slight differences in ACSF and internal solutions. However, we found that proportions of firing neurons increased from 20 to 40 DPH and then decreased at 60 DPH, while that of silent neurons decreased from 20 to 40 DPH and then increased at 60 DPH in normally reared male and female juveniles. Considering previous studies, increasing proportions of silent neurons at 60 DPH might be one of the trajectories of development towards adulthood. Though we cannot identify neuron type perfectly, our trial for cell identification with virus expression, neurophysiological and morphological study suggest firing neurons included both excitatory and inhibitory neurons. Changing excitatory and inhibitory balance may work for neuronal circuit plasticity. Further pharmacological studies are needed to determine whether this developmental change depends on the intrinsic membrane properties of neurons or synaptic connections. Interestingly, in juveniles that had been isolated from adult tutor song experiences (but not from juvenile singing of siblings), which had an extended sensory learning period, neurons exhibited a different timeline for developing firing properties. Proportions of silent neurons decreased from 40 to 60 DPH, which was later than in normally reared juveniles, and then stayed low until 80 DPH. These findings suggested the delayed development of neurophysiological properties with auditory experience deprivation. Delayed development of neurophysiological properties with auditory isolation was shown in the song system in males (Livingston et al., 2000). The NCM is thought to be one of the brain loci for tutor song auditory memory storage, as it shows neuronal circuit modifications upon auditory experience with tutor songs. Although the recording ages were different from this study, a previous report showed that acute auditory experiences in adults altered bursting glutamatergic neurotransmission after blockade of GABAergic transmission, with a small difference between sexes (Dagostin et al., 2012). This suggests that auditory-experience-dependent neuronal circuit modification occurs in the NCM. A recent study in another auditory cortical area, the CMM, which also has been suggested to store tutor song memory, shows that the proportions of phasic-firing neurons changes, depending on experience and sex (Chen and Meliza, 2020). Neuronal circuits in the NCM were modified in both males and females, but perhaps differently during the sensory learning period, which probably depends on auditory tutor song experiences. While isolation affected proportions of firing neurons similarly in males and females, proportions of burst type neurons differed between males and females. This suggests that while auditory experience shapes neuronal circuits of both males and females in the NCM, the mechanism by which this occurs may differ between the sexes. In this study, juveniles were isolated from adult tutor songs, but not from sibling immature songs and female vocalizations. A previous study suggests that non-song vocalizations affect the auditory responsiveness of NCM neurons differently in males and females (Phan and Vicario, 2010). While it is still controversial, isolation or altering auditory experiences cause abnormal song discrimination in males but have no effects on females (Campell and Hauber, 2009; Maul et al., 2010; reviewed Woolley, 2012). While the NCM is thought to be involved in song learning in males (Bolhuis et al., 2000; Terpstra et al., 2004; London and Clayton, 2008; Yanagihara and Yazaki-Sugiyama, 2016), it has also been reported as an auditory area in females, and auditory experiences alter the responsiveness of the female NCM as well (Yoder et al., 2015; Diez et al., 2019; Inda et al., 2020). Auditory experiences with tutors may also cause different neuronal circuit modifications in juvenile males and females. Collectively, these findings suggest that auditory experiences with tutor songs modulate neuronal circuit connectivity in the NCM, leading to modifications in neuronal firing activities of each neuron in both males and females, but in different manners.

The timing of the critical period depends on the maturation of GABA inhibitory functions, and sensory deprivation delays the opening and closing of the critical period (Hensch, 2004). Auditory deprivation in the early sensory period delays the development of NMDA currents in the brain area that is necessary for song learning (White et al., 1999; Livingston et al., 2000). A recent study reported that populations of parvalbumin-positive neurons are altered during the sensory learning period, and that age and rearing condition (auditory isolation) had significant interactions (Gogola et al., 2019). Our previous study showed that response selectivity decreased after local GABA blockade, suggesting the developmental recruitment of GABA inhibitory circuits (Yanagihara and Yazaki-Sugiyama, 2016). GABA inhibitory activities shape the auditory responses of NCM neurons (Pinaud et al., 2008). Experience-dependent GABA current development has been shown in a variety of sensory cortexes, such as V1 and A1 (Fagiolini and Hensch, 2000; Takesian et al., 2018). Our study suggested that the proportions of firing neuron change during development and that firing neurons include inhibitory neurons. While with the current study we could not identify each cell type with SFR, spike shape, morphology, or these combinations. Further studies to identify neuronal cell types would illuminate the effects of auditory experience on inhibitory circuits in the NCM.

In this study, we examined NCM spontaneous neural activity from brain slices and tracked its development during the sensory learning period for the first time. Additional studies on NCM neuronal circuit development and its dependence on experience will further explain what happens in the NCM, depending upon early auditory experiences and sex.

## Data Availability Statement

All datasets presented in this study are included in the article/Supplementary Material.

## Ethics Statement

The animal study was reviewed and approved by The animal care committee at Okinawa Institute of Science and Technology (OIST) Graduate University.

## Author Contributions

TK and YY-S designed the experiments. TK performed the experiments and analyzed the data. YM constructed and made the AAVs. TK and YY-S wrote the article. All authors contributed to the article and approved the submitted version.

## Conflict of Interest

The authors declare that the research was conducted in the absence of any commercial or financial relationships that could be construed as a potential conflict of interest.
