# Supplementary material for: Early Auditory Experience Modifies Neuronal Firing Properties in the Zebra Finch Auditory Cortex
Source: Front Neural Circuits. 2020 Oct 8;14:570174. doi: 10.3389/fncir.2020.570174 (PMC7578418; doi:10.3389/fncir.2020.570174)
Supplement: Supplementary file 1 [file Data_Sheet_1.PDF]

# *Supplementary Material*

## Supplemental Figure 1

**A:** Average spontaneous firing rate (SFR) of firing neurons recorded on the brain NCM brain slice from male and females normally raised juveniles at 20, 40 and 60 DPH (mean ± sem). **B:** Plots of resting membrane potential (Vm) against SFR of each firing neuron recorded on the brain NCM brain slice from male and female normally raised juveniles at 20, 40 and 60 DPH. **C:** Histograms of the number of NCM neurons as a function of their SFR in normally raised male and female juveniles at 20, 40 or 60 DPH which are color coded for each bird (the same color blocks denote the neurons recorded from the same birds). The neurons in the first column in the histogram (SFR = 0) denoted silent neurons. (bird: N = 5, 17 and 12 for males at 20, 40 and 60 DPH, 11, 12 and 10 for females at 20, 40 and 60 DPH birds, respectively.)

## Supplemental Figure 2

**A:** Plots of resting membrane spontaneous firing rate (SFR) against spike half width of each firing neuron recorded on the brain NCM brain slice from male and female normally raised juveniles at 20, 40 and 60 DPH. **B:** Plots of resting membrane spontaneous firing rate (SFR) against spike half width of morphologically identified with *post hoc* staining firing neurons recorded on the brain NCM brain slice from male and female normally raised juveniles at 20, 40 and 60 DPH, and from the mRFP-expressing neurons from 60DPH male and female juveniles injected with AAV-mDlx-mRFP. C: Plots of SFR against burst rate (# of burst during recording / s) of each burst type neuron recorded on the brain NCM brain slice from male and female normally raised juveniles at 20, 40 and 60 DPH. **D:** Confocal images of the recorded spiny (top) and aspiny (bottom) neurons and their spike shape.

## Supplemental Figure 3

Representative example of traces of the NCM neurons for monitoring spontaneous firing rate (**A**) and measuring input resistance (Rin) with hyperpolarizing pulse current injection (slope of the voltage responses to -10 to -50 pA of the current injection) (**B**).

## Supplemental Figure 4

**A:** Proportions of firing neurons recorded from male and female isolated juveniles at 20, 40, 60 and 80 DPH. **B:** Plots of resting membrane potential (Vm) against SFR of each firing neuron recorded on the brain NCM brain slice from male and female isolated juveniles at 20, 40 and 60 DPH. **C:** Plots of resting membrane spontaneous firing rate (SFR) against spike half width of morphologically identified with *post hoc* staining firing neurons recorded on the brain NCM brain slice from male and female isolated juveniles at 20, 40 and 60 DPH. **D:** Average spontaneous firing rate (SFR) of firing neurons recorded on the brain NCM brain slice from male and females isolated juveniles at 20, 40 and 60 DPH (mean ± sem). **E:** Histograms of the number of NCM neurons as a function of their

spontaneous firing rate (SFR) in isolated male and female juveniles at 20, 40 or 60 DPH which are color coded for each bird (the same color blocks denote the neurons recorded from the same birds). The neurons in the first column in the histogram (SFR = 0) denoted silent neurons. (N = 2, 7, 5 and 14 for males at 20, 40 and 60 DPH, 3, 9, 7 and 6 for females at 20, 40 and 60 DPH birds, respectively.)

Supplemental table 1: Estimated mean SFR by using a generalized linear model with a Poisson distribution with its 95% confidence interval.

| **Normal** | | **Male** | | | | **Female** | |
|---|---|---|---|---|---|---|---|
| | **20DHP** | **40DPH** | **60DPH** | | **20DHP** | **40DPH** | **60DPH** |
| **Mean** | 1.30 | 0.96 | 0.71 | | 1.96 | 1.45 | 1.07 |
| **95% CI bottom** | 0.99 | 0.76 | 0.52 | | 1.55 | 1.20 | 0.80 |
| **95% CI top** | 1.72 | 1.22 | 0.98 | | 2.47 | 1.76 | 1.44 |
| Generalized linear regression model: log(y) ~ 1 + x1 + x2, Distribution = Poisson | | | | | | (x1: sex, x2: age) | |

| **Isolate (Male/Female)** | **20DHP** | **40DPH** | **60DPH** |
|---|---|---|---|
| **Mean** | 1.95 | 1.52 | 1.19 |
| **95% CI bottom** | 1.51 | 1.30 | 0.95 |
| **95% CI top** | 2.53 | 1.79 | 1.49 |

Generalized linear regression model: log(y) ~ 1 + x2, Distribution = Poisson (x2: age)
