# Supplementary figures and images for: Early Auditory Experience Modifies Neuronal Firing Properties in the Zebra Finch Auditory Cortex
Source: Front Neural Circuits. 2020 Oct 8;14:570174. doi: 10.3389/fncir.2020.570174 (PMC7578418; doi:10.3389/fncir.2020.570174)

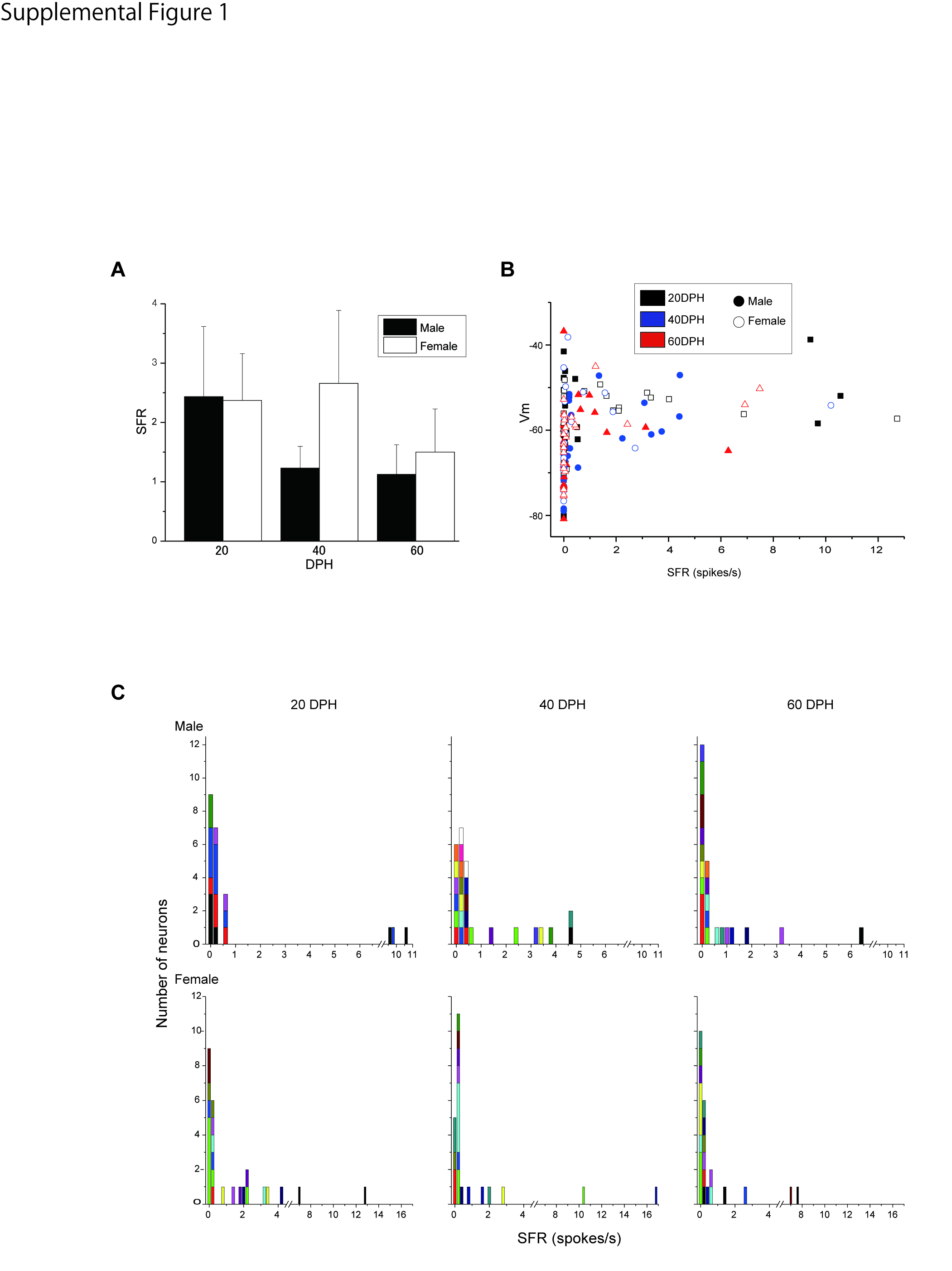

Supplement: Supplementary file 2 [file Image_1.TIF]

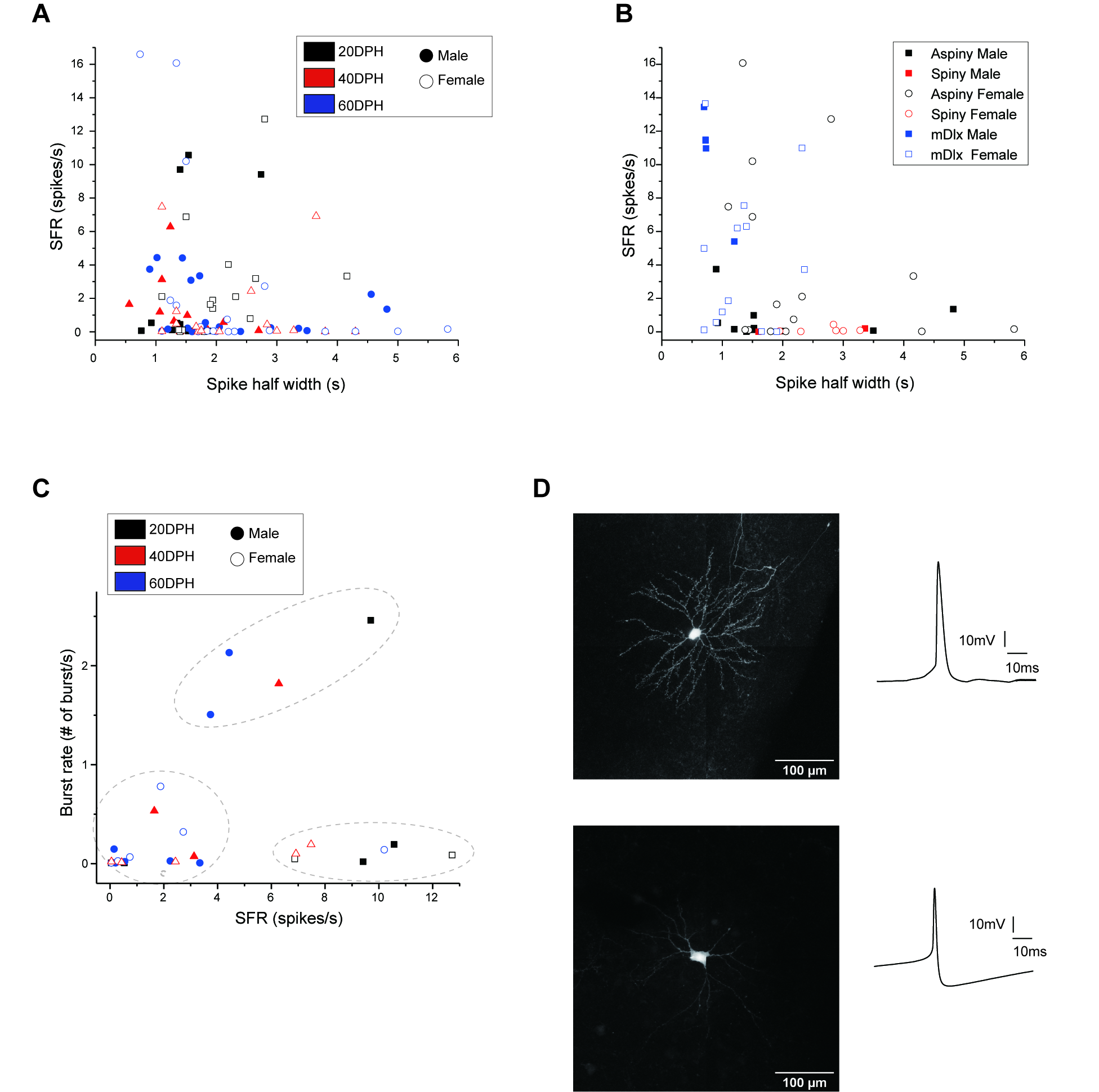

Supplement: Supplementary file 3 [file Image_2.TIF]

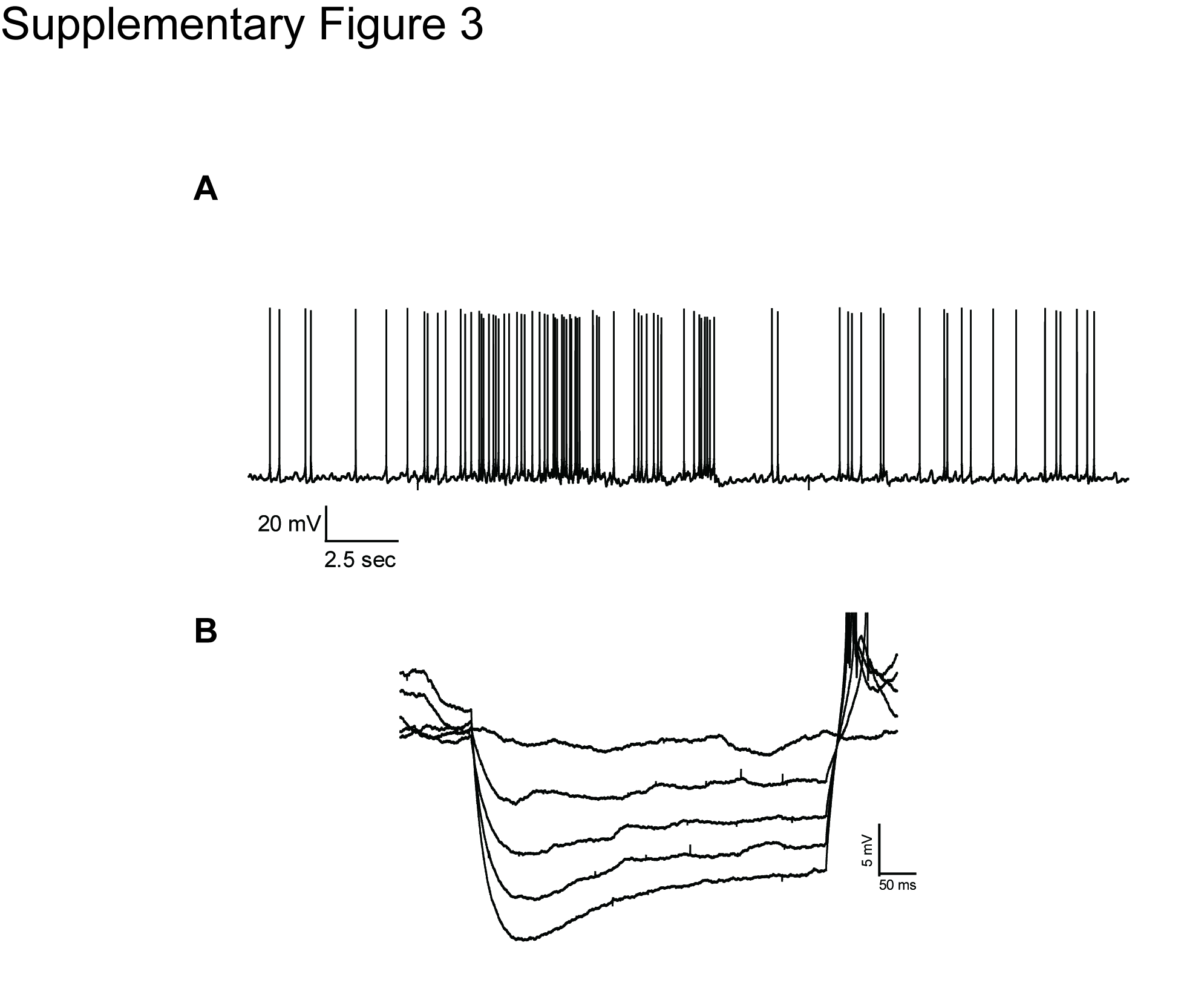

Supplement: Supplementary file 4 [file Image_3.TIF]

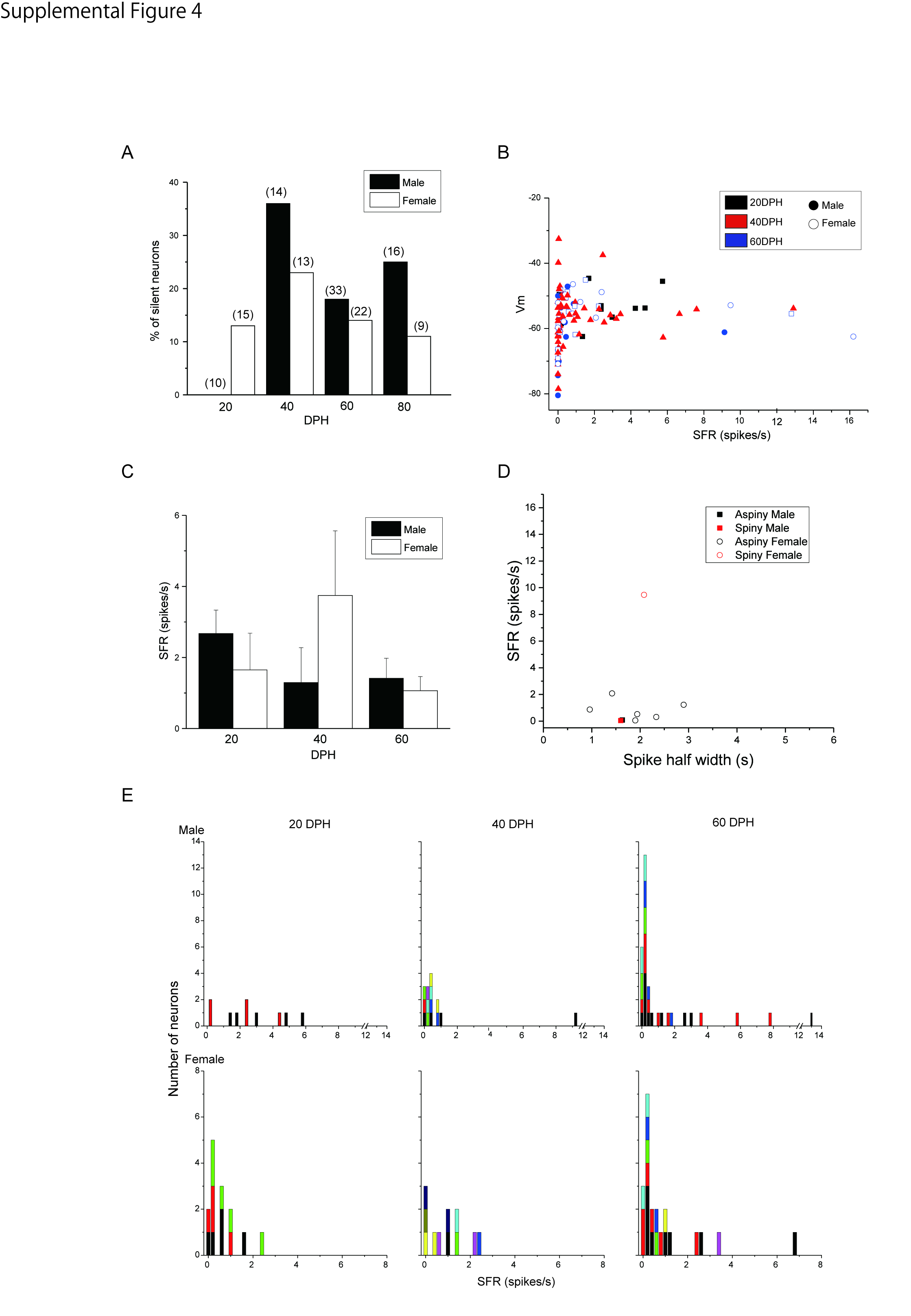

Supplement: Supplementary file 5 [file Image_4.TIF]
